# Optical and structural characterization of oleic acid-stabilized CdTe nanocrystals for solution thin film processing

**DOI:** 10.3762/bjnano.5.100

**Published:** 2014-06-20

**Authors:** Claudio Davet Gutiérrez-Lazos, Mauricio Ortega-López, Manuel A Pérez-Guzmán, A Mauricio Espinoza-Rivas, Francisco Solís-Pomar, Rebeca Ortega-Amaya, L Gerardo Silva-Vidaurri, Virginia C Castro-Peña, Eduardo Pérez-Tijerina

**Affiliations:** 1 Centro de Investigación en Ciencias Físico Matemáticas, Facultad de Ciencias Físico Matemáticas, Universidad Autónoma de Nuevo León, Av. Universidad s/n. Ciudad Universitaria, 66451 San Nicolás de los Garza, Nuevo León, México; 2 Sección de Electrónica del Estado Sólido, Centro de Investigación y de Estudios Avanzados del Instituto Politécnico Nacional, Av. Instituto Politécnico Nacional 2508, Col. San Pedro Zacatenco, 07360 México, D. F., México; 3 Programa de Doctorado en Nanociencias y Nanotecnología, Centro de Investigación y de Estudios Avanzados del Instituto Politécnico Nacional, Av. Instituto Politécnico Nacional 2508, Col. San Pedro Zacatenco, 07360 México, D. F., México

**Keywords:** cadmium telluride, Raman spectroscopy, semiconductor nanocrystals, transmission electron microscopy, X-ray diffraction

## Abstract

This work presents results of the optical and structural characterization of oleic acid-stabilized cadmium telluride nanocrystals (CdTe-NC) synthesized by an organometallic route. After being cleaned, the CdTe-NC were dispersed in toluene to obtain an ink-like dispersion, which was drop-cast on glass substrate to deposit a thin film. The CdTe-NC colloidal dispersion as well as the CdTe drop-cast thin films were characterized with regard to the optical and structural properties. TEM analysis indicates that the CdTe-NC have a nearly spherical shape (3.5 nm as mean size). Electron diffraction and XRD diffraction analyses indicated the bulk-CdTe face-centered cubic structure for CdTe-NC. An additional diffraction line corresponding to the octahedral Cd_3_P_2_ was also detected as a secondary phase, which probably originates by reacting free cadmium ions with trioctylphosphine (the tellurium reducing agent). The Raman spectrum exhibits two broad bands centered at 141.6 and 162.3 cm^−1^, which could be associated to the TO and LO modes of cubic CdTe nanocrystals, respectively. Additional peaks located in the 222 to 324 cm^−1^ range, agree fairly well with the wavenumbers reported for TO modes of octahedral Cd_3_P_2_.

## Introduction

Currently colloidal chemistry is a promising technique in materials science research due to its simplicity, low cost, and its capability to process nearly-monodisperse nanocrystals of a variety of semiconductor materials at low temperatures and using non sophisticated equipments [1–2]. It has been extensively used to prepare binary [3–5], ternary [6–8] and quaternary [9–10] semiconductors for applications in quantum effect based devices [11] such as light emitting diodes [12], biolabeling [13], thermoelectric generators [14] and thin film solar cells [15]. In solar cell technology, colloidal nanocrystals hold promise for producing cheap solar cells with improved conversion efficiency by using quantum effects such as multiple exciton generation [16]. In the last years, nanostructured semiconductors have proved to be useful as raw material for solution-processable electronic technologies. The capping of semiconductor nanocrystals by organic ligands, provides the control of composition, size, shape and crystal structure, which make them very attractive in this technological area. Thin films deposition techniques such as drop-casting [17], screen printing [18], and ink jet printing [19], are currently used to fabricate electronic devices on great variety of substrates, either rigid or flexible, looking for preserve their size effects [20–22]. All of these techniques need ink-like stable dispersions comprising functionalized nanomaterials dispersed in a convenient solvent. Drop-casting offers a cost efficient production of photovoltaic semiconductor thin films. Nevertheless, depending on the dispersion chemistry and on the required thin film optoelectronic properties, post-deposition complex processes are needed. Guo et al. [23] deposited CuInSe_2_ (CIS) thin films for solar cells starting from oleylamine-stabilized CIS nanocrystals. An additional annealing at 500 °C was needed to obtain suitable CIS films for photovoltaic applications. Jasieniak et al. [24] deposited photovoltaic cadmium telluride (CdTe) thin films by using pyridine-capped CdTe nanocrystals. In their approach, CdTe nanocrystals were deposited from solution by a layer-by-layer process with subsequent annealing per layer at 300, 350 and 400 °C. On the other hand, oleic acid is a green organic ligand, which has been successfully used in preparing a great variety of colloidal materials [15,25–26], including CdTe quantum dots [27–29].

This work presents our early results on the elaboration of CdTe-based inks for potential uses in solution-processable thin film solar cells. The CdTe-NC were prepared by colloidal chemistry using an organic–inorganic reaction [30]. The product comprised toluene-dispersed oleic acid-stabilized CdTe-NC and cadmium phosphide (Cd_3_P_2_) as a secondary phase. Oleic acid was chosen because it enabled us to manipulate the CdTe nanocrystals under ambient conditions, and to use them to deposit CdTe thin films that were free of cadmium tellurate or tellurium oxide. The CdTe nanocrystals and thin films were characterized by TEM, X-ray diffraction, UV–vis spectroscopy and Raman spectroscopy.

## Results and Discussion

### TEM analysis

**Figure 1:**
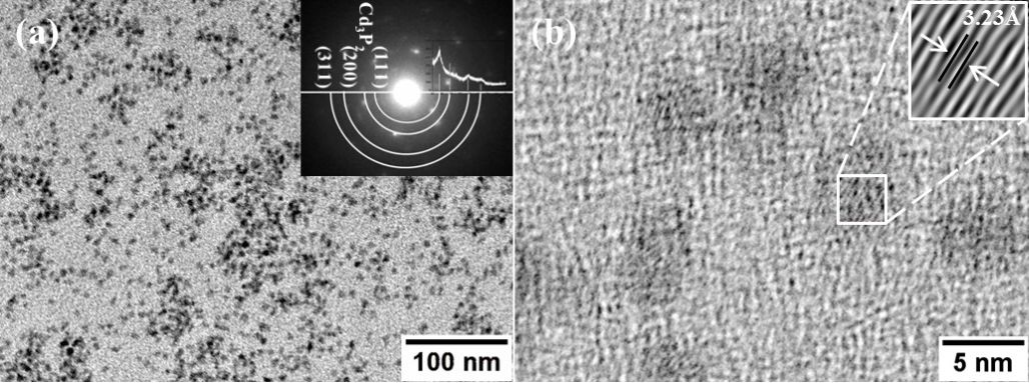
a) TEM micrograph of CdTe nanocrystals, the inset shows the indexed electron diffraction pattern, b) TEM image of CdTe nanocrystals used to determinate the lattice parameter. The inset shows the 3.23 Å (200) lattice parameter.

In general, well crystallized CdTe colloids were obtained, as shown in Figure 1b. We were unable to assess the size and shape of the samples. Although the absorbance spectrum displays a sharp excitonic peak indicating a nearly monodispersed nature of our samples, the degree of monodispersity could not be evaluated from the TEM micrograph. The TEM image of Figure 1a shows CdTe-NC with a size of 3.5 nm, the electron diffraction pattern (inset Figure 1a), indicated the face-centered cubic phase for CdTe as reported by Talapin et al. [31]. The electron diffraction pattern was indexed following the method reported elsewhere [32]. In addition, an extra phase was disclosed; it was attributed to the Cd_3_P_2_ impurity phase, which crystalizes in the octahedral structure. The above described features could be corroborated by XRD and Raman spectroscopy (see below).

**Figure 2:**
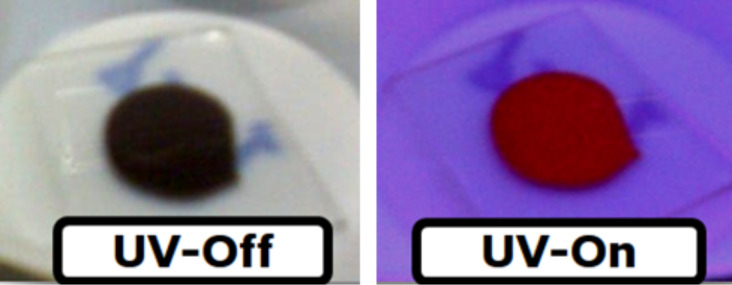
CdTe deposition with and without UV illumination under ambient conditions.

Figure 2 shows a CdTe deposition with and without UV illumination. We further note that the nanocrystal luminescence under ambient conditions is preserved.

### X-ray diffraction analysis

**Figure 3:**
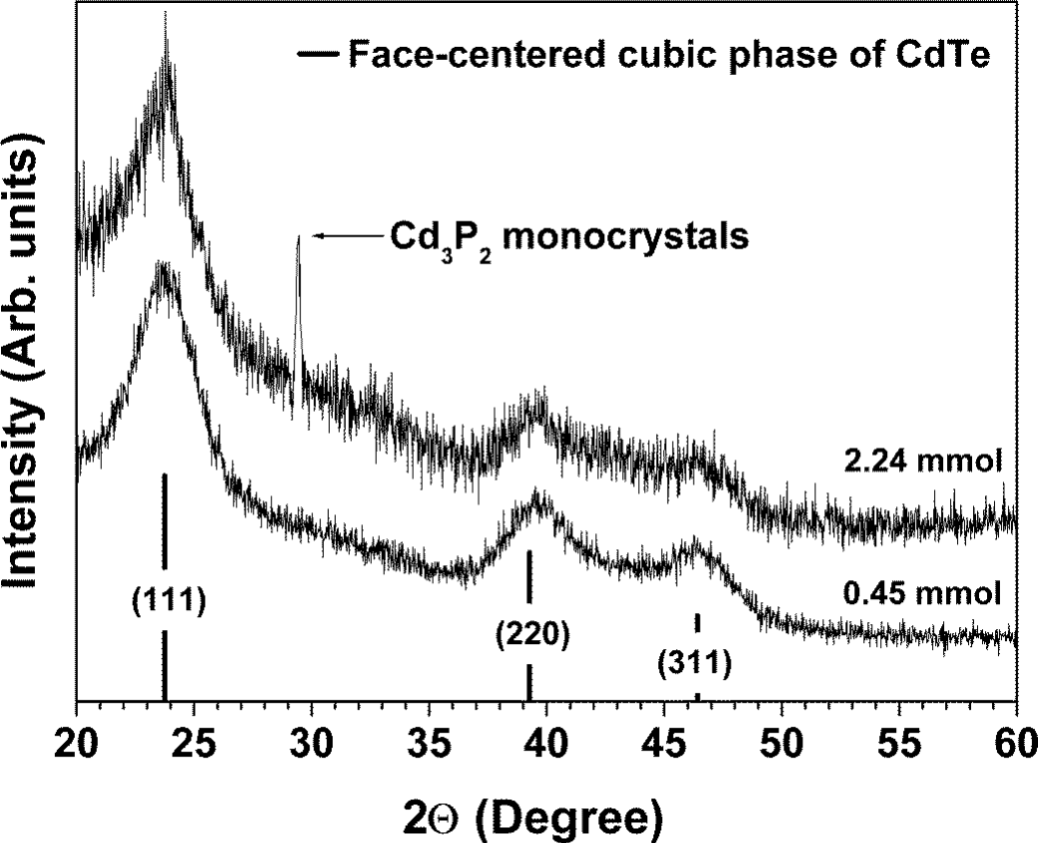
X-ray diffraction pattern of deposited CdTe nanocrystals.

The structural properties of the prepared samples were evaluated by XRD and Raman spectroscopy, using CdTe thin films deposited on glass substrates (see Figure 2). As mentioned above, XRD and Raman measurements corroborated the phase composition of the colloids. Figure 3 shows the X-ray diffraction patterns of deposited CdTe-NC at concentrations of 2.24 and 0.45 mmol trioctylphosphine (TOP), respectively. The diffraction patterns display the typical low intensity and broad peaks of a nanosized material. The peaks at 23.7, 39.3 and 46.6°, could be ascribed to (111), (220) and (311) diffraction lines of the face-centered cubic structure of CdTe [33–34]. The narrow peak at 29.5° appearing at a concentration of 2.24 mmol TOP could be attributed to the octahedral phase of Cd_3_P_2_ [35], so testifying the electron diffraction results. It is also observed that Cd_3_P_2_ disappeared at the low TOP concentration (Figure 3). The crystal size, as determinated by using the Debye–Scherrer formula, was 3.7 nm, which is in good agreement with the value ascertained by TEM. The Debye–Scherrer formula is [36]:


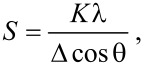


where *K* is the Scherrer constant, taken as 0.9 for cubic crystal structures, λ is the X-ray wavelength, Δ is the full width at half maximum (FWHM) of the diffraction peak and θ is the Bragg angle.

### Raman spectroscopy

**Figure 4:**
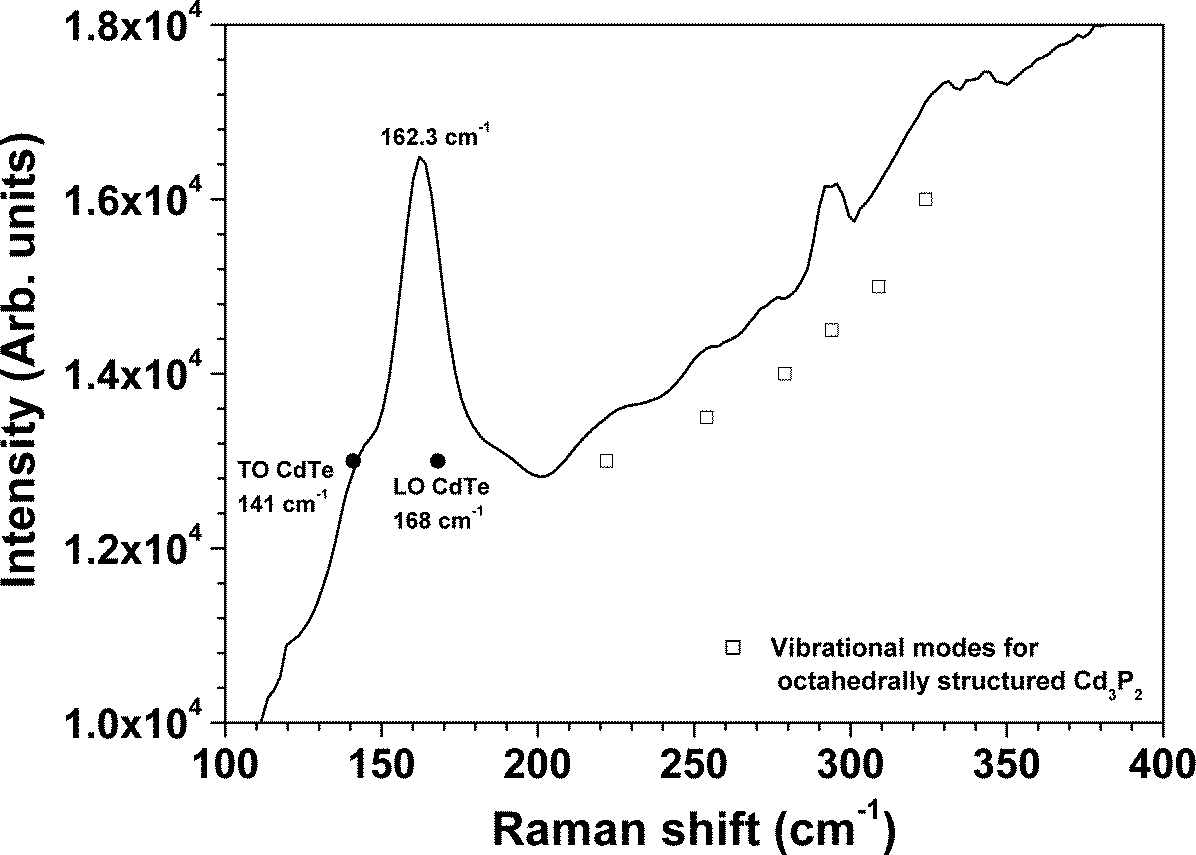
Raman spectrum of drop-cast CdTe nanocrystals measured with a 632 nm He–Ne laser.

Raman spectroscopy is a valuable technique to assess the structure of semiconductors also at the nanometer-scale. In particular, Raman spectroscopy is sensitive to size effects, because like the excitons, phonons experience quantum confinement effects depending on the crystal size. In CdTe, the quantum confinement effect is revealed by the broadening and red shifting of the LO mode [37]. Figure 4 shows the cubic CdTe Raman peaks at 142 and 162.3 cm^−1^ corresponding to TO and LO modes [38], respectively. It is seen that, the LO mode red-shifts by 5.7 cm^−1^ with respect to bulk CdTe [38]. On the other hand, all Raman peaks in the range between 200 and 350 cm^−1^ belong to octahedral Cd_3_P_2_ [35], as previously stated by TEM and XRD.

In addition, we have used the Richter–Wang–Law model [37,39], to calculate the crystallite size by fitting the LO peak. The obtained value was 3.4 nm, which is in good agreement with that calculated by TEM and XRD. Interestingly, the CdTe crystallites preserve their size when deposited in the glass substrate, so testifying the suitable oleic acid stabilization. In regarding the Cd_3_P_2_ formation, it is thought that it was originated by reacting cadmium and phosphor ions, with the latter being provided by the TOP decomposition. Because as reported by Chen et al. [40] and Henkes et al. [41], the high reactivity of transition metals promotes the P–C cleavage and the release of the phosphor ions. Surprisingly, the Cd_3_P_2_ formation depends on the TOP concentration, because as indicated in Figure 3, for TOP concentration lower than 2.4 mmol, impurity-free CdTe colloids were obtained.

### Optical characterization

**Figure 5:**
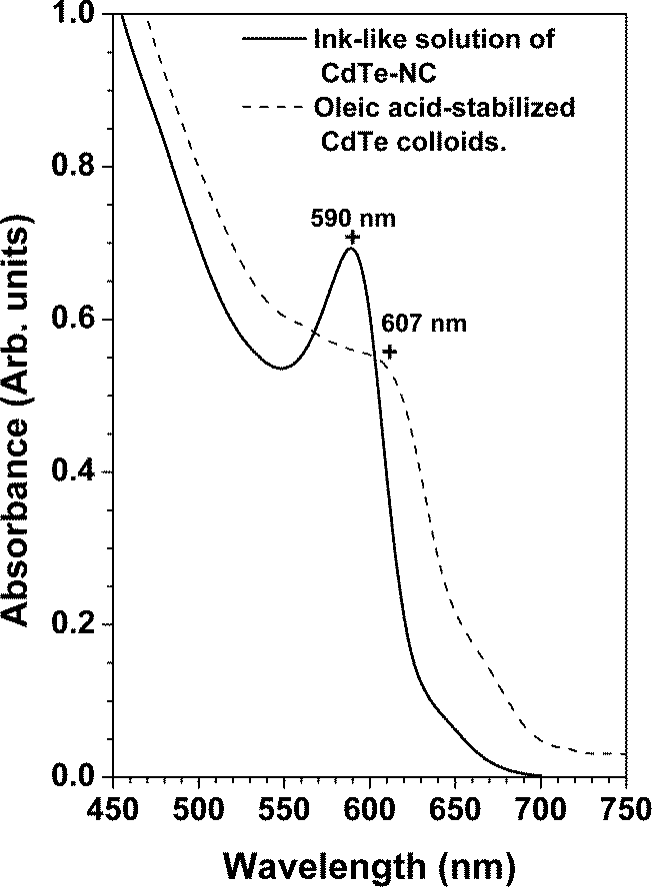
Absorbance spectra of the colloidal solution and CdTe-NC processed by size-separation and re-dispersed in toluene.

According to Figure 2, our CdTe colloids display a strong red luminescence, indicating quantum confinement in the nanosized CdTe crystals. It is known that quantum confinement can be observed for crystallite sizes lower than the exciton Bohr radius of the bulk counterpart. In the case of CdTe, the reported parameter is 6.8 nm [42], whilst the CdTe crystal size was 3.5 nm, as estimated from TEM and XRD measurements. The absorbance spectra shown in Figure 5 exhibit a well-defined peak in the visible wavelength range. This peak corresponds to the band-to-band transition, since it is the most likely transition under the quantum confinement regime [43]. It is worthy to note that CdTe nanocrystals dispersed in toluene showed a narrow absorption peak (or excitonic peak) around 590 nm, suggesting a narrow size distribution for our samples. Nevertheless the exciton peak disappeared once the CdTe colloid was drop-cast on the glass substrates Figure 5 shows the absorbance spectra of CdTe-NC of the colloidal solution and of a liquid sample processed by the size-separation technique. The effect of size-separation can be observed by considering the maximum absorption peak. The excitonic peak is narrower than that the of the colloid and nearly symmetric, similar to a nearly monodispersed sample. Additionally, the maximum absorbance has shifted from 607 nm to 589 nm arguing for a lower size dispersion of the CdTe-NC. However, this result imposes a technological limitation in the colloid application in a thin-film device due to strong stabilization of oleic acid, which is nonconductive and separates the nanocrystals with spacings of about 0.7 nm and thus impedes their electrical contact [15].

## Conclusion

Oleic acid-stabilized CdTe nanocrystals were prepared by colloidal chemistry. A toluene dispersible product comprising CdTe and Cd_3_P_2_ as impurity was obtained. The ink-like dispersion was successfully to deposit thin films on glass substrate. It was demonstrated that the phase composition depends strongly on the TOP concentration, TOP concentration lower than 2.24 mmol produced free Cd_3_P_2_ CdTe colloids.

## Experimental

**Materials:** cadmium oxide powder ≈1 μm (CdO, 99.5% Aldrich); tellurium powder (200 mesh 99.8% Aldrich); oleic acid (OA, technical grade 90% Aldrich); *n*-trioctylphosphine (TOP, technical grade 90% Aldrich); 1-octadecene (1-ODC, technical grade 90% Aldrich).

**Synthesis of CdTe nanocrystals:** Colloidal CdTe-NCs were synthesized via an organic–inorganic route using oleic acid as the stabilizer and 1-octadecene as the solvent. The tellurium precursor was prepared by dissolving tellurium powder (12.8 mg, 0.1 mmol) in a previously prepared 1-octadecene (4 mL)/TOP (1 mL, 2.24 mmol or 0.2 mL, 0.45 mmol) mixture. The solution was heated to 120 °C for 15 min, under N_2_ atmosphere and vigorous magnetic stirring. The TOP–Te solution was maintained under inert atmosphere until its use. Cadmium oleate was used as cadmium precursor, which was prepared also under N_2_ atmosphere by mixing 25.6 mg (0.2 mmol) of cadmium oxide and 0.5 mL (0.315 mmol) of oleic acid in 10 mL of 1-octadecene. Reagents were mixed with vigorous magnetic stirring at 210 °C until the complete dissolution of CdO powder. Subsequently, the TOP–Te solution was transferred through flexible tubing by using N_2_ flux and the colloid temperature was reduced to 190 ± 2 °C. This value was maintained as reflux temperature.

**Sample preparation to optical and structural characterization:** Initially, the CdTe-NC were processed by a size separation technique. On a colloidal solution, ethanol aliquots were dropped and the flocculated solid phase was removed from solution, rinsed also with ethanol and dried with N_2_. This process was repeated until the solid phase remained completely attached to the flask bottom. Finally, nanocrystals were dispersed with 2–4 mL toluene and deposited on glass substrates via drop-casting technique without annealing. For absorbance characterization, a liquid sample processed by the size-separation technique was analyzed and for Raman measurements, the same sample but deposited by dripping on glass substrate was studied.

**TEM characterization:** Shape, size and crystal structure of CdTe-NC were determined by transmission electron microscopy (TEM) using a JEOL 2010 microscope. For TEM characterization, the nanocrystals solution were dropped on 300 mesh Lacey carbon grid and dried under laboratory conditions. X-ray diffraction (XRD) measurements were done on depositions by using a Bruker AXs D8 Discover diffractometer at normal incidence, using Cu Kα radiation (λ = 0.1506 nm). Absorbance of CdTe colloids and CdTe nanocrystals re-dispersed in toluene were carried out at room temperature by using a Shimadzu 2401 UV–vis spectrophotometer. Raman spectroscopy was carried out also on drop-cast samples by using a Horiba Jobin-Yvon system with an Olympus BX40 microscope, employing the 632 nm He–Ne laser wavelength.
